# Comparison of the effects of low dose interferon and high dose interferon on reduction of the number and size of plaques in patients with Multiple Sclerosis: A historical cohort

**Published:** 2017-01-05

**Authors:** Payam Khomand, Ghobad Moradi, Behrooz Ahsan, Setareh Abtahi

**Affiliations:** 1 Department of Neurology, School of Medicine, Kurdistan University of Medical Sciences, Sanandaj, Iran; 2 Social Determinants of Health Research Center, Kurdistan University of Medical Sciences, Sanandaj, Iran; 3 Departments of Neurology, Tohid Hospital, Kurdistan University of Medical Sciences, Sanandaj, Iran

**Keywords:** Multiple Sclerosis, High Dose Interferon, Low Dose Interferon, Magnetic Resonance Imaging

## Abstract

**Background:** This study was performed to compare the effects of low dose interferon beta-1 (IFN-β-1) (CinnoVex, 30 mcg) and high dose IFN-β-1 (REBIF, 44 mcg) on the reduction of the number and size of plaques in magnetic resonance imaging (MRI) in patients with multiple sclerosis (MS).

**Methods:** This historical cohort study, which was performed in 2014 in Sanandaj (western part of Iran). 43 MS patients in two groups were investigated. The first group, which included 19 patients, was treated using high dose IFN (44 mcg) and the second group, which was consisted of 24 patients, was treated using low dose IFN (30 mcg). Patients’ data were collected and analyzed by the Stata version 11 software; the analyses were performed using statistical t-test, chi-square test, Fisher test, and logistic regression.

**Results:** Both drugs were effective in controlling active demyelinating plaque and in preventing plaque activation (P = 0.633). The impact of both drugs in the reduction of the number and size of plaques was evaluated. Based on the results of the MRI, high dose IFN therapy was more effective than the low dose IFN drugs and had a better performance in terms of reducing the number of plaques and in stop-and-recovery (P = 0.039), as well as in reducing the plaque size (P = 0.050).

**Conclusion:** The high dose IFN therapy was more effective than the low dose IFN therapy in reducing the number and size of brain plaques in patients with relapsing-remitting MS (RRMS).

## Introduction

Multiple sclerosis (MS) is a chronic, recurrent inflammatory and demyelinating disease which involves the central nervous system. It mostly affects young women and causes disabilities in patients.^1^ There are several different types of MS including relapsing-remitting form of MS (RRMS), primary progressive MS, secondary progressive MS, and isolated clinical syndrome.^2^

The first line of treatment for RRMS is consisted of interferon beta (IFN-β) and is glatiramer acetate.^3^ They have a good effect on reducing relapse and a variety of disabilities and on magnetic resonance imaging (MRI) criteria. Effect of IFN-β for the treatment of RRMS has been proved. IFN therapy can make its effect via its anti-proliferative effects and reduces the permeability in the blood–brain barrier.

MRI has a high capacity for early diagnosis of MS, particularly if the clinical diagnosis is uncertain, monitoring of treatment, evaluation of disease progression, and response to treatment.^4^

Abnormalities in brain MRI are observed in more than 95% of newly diagnosed patients. There are 5-10 new or large plaques enhanced with gadolinium; on the other hand, T2 lesions show attacks in any patient with RRMS.^5^ Brain MRI can show the areas of edema, demyelination, damaged axons, gliosis, and repair of myelin in areas with high signal in T2.^5^^,^^6^

Beta IFN compounds include beta IFN-β-1a (Avonex) or low dose IFN and high dose IFN-β-1a (REBIF) and beta IFN-β-b called (Betaseron).^7^^-^^10^

IFN-β1a lowers the rate of attacks in MS patients by 33%.

High dose IFN-β-1-a (REBIF) is one of two available formulations of IFN-β1. This drug is used and injected subcutaneously at doses of 22 and 44 µg 3 times a week.^11^ Low dose IFN-β-1a (Low dose IFN) is prescribed for intramuscular injection at a dose of 30 mg once a week low dose IFN (CinnoVex is the commercial name of IFN-β-1a and it is manufactured in Iran as the world’s third largest manufacturer in the market). It is a biosimilar or biogeneric of Avonex drug.^12^

Some studies have shown that IFN-β compounds can reduce MS attacks, brain atrophy, and the number and volume of brain lesions.^3^^,^^11^ Other studies have suggested the better effects of low dose IFN-β-A and high dose (REBIF) in reducing plaques in MRI compared with placebo.^13^

Although the low dose IFN drug is used abundantly by MS patients in Iran, few study has been conducted on the effects of the drug (especially CINOVEX) on MS patients in terms of reducing the number of plaques or to compare it with high dose IFN drugs. Furthermore, we assume this survey may be a view about Kurdish patients with MS and an effect of Iranian products of IFN-β-1-a (CinnoVex) on them, which is an important medical issue in our area. 

Accordingly, this study examines the impact and efficacy of low dose IFN (CinnoVex) on reducing the number of MRI plaques in MS patients and compares it with high dose IFN (REBIF).

## Materials and Methods

This study was a historical cohort and it was conducted on patients with RRMS who were under the treatment with low dose IFN drugs (CinnoVex) or high dose IFN (REBIF); the patients had a profile in the Clinic of Kurdistan University of Medical Sciences or in Sanandaj MS Society, Iran. The study was conducted in 2014. 

Although clinical trial with randomization is the best way to test the hypothesis of this study, because of budgetary limitations and the long duration of the project, it became difficult for the researchers to use this method. 

IFN-β-1a is sold under the trade names Avonex (Biogen) and Rebif (Merck Serono), (Pfizer); CinnoVex (CinnaGen) is biosimilar of Avonex. Rebif, it is co-marketed by Merck Serono and Pfizer in the US. 

CinnoVex is the trade name of recombinant IFN-β-1-a, which is manufactured as biosimilar/biogeneric in Iran. It is produced in a lyophilized form and sold with distilled water for injection. CinnoVex was developed at the Fraunhofer Institute in collaboration with CinnaGen. Dosage of both drugs in this study was 44 mcg (REBIF) and 30 mcg (CinnoVex), respectively.

According to inclusion criteria patients with the following features were included in the study: men and women aged between 18 and 50 years, patients who had been under treatment with low dose or high dose IFN for at least a year before the study, patients who were not pregnant and did not breastfeed a child, patients who were using a reliable contraception method, patients who were identified as RRMS according to the latest amendments to the Revise McDonald 2010 criteria, patients whose Expanded Disability Status Scale (EDSS) was ≤ 5.5, patients who were not concurrently taking drugs that had interference with low dose or high dose IFN, and patients who consented and were able to cooperate until the end of the project. 

**Table 1:** Association of Interleukin 6 (IL-6) level with National Institutes of Health Stroke Scale (NIHSS), modified Rankin Scale (mRS) and other infarcts

**Variables**	**Low dose IFN **	**High dose IFN**	**P**
Sex [n (%)]			
Male	4 (17)	4 (21)	0.714*
Female	20 (83)	15 (79)	
Age (year) (Mean)	33.58	29.84	0.070**

* Chi-square,

** t-test. IFN: Interferon

Exclusion criteria included the following: patients whose symptoms were the likely signs of other diseases other than MS, patients who had fully transverse myelitis or bilateral optic neuritis, patients with clinically isolated syndrome, and patients who had enhanced plaque in the initial MRI.

With regarding difference between 2 groups based on the effects of outcome, it was equal to 40% and p_1_ = 30% also, with regarding p_1_ = 70% with 5% alpha and beta 20% sample size (based on the below formula) was 21 patients for each group.


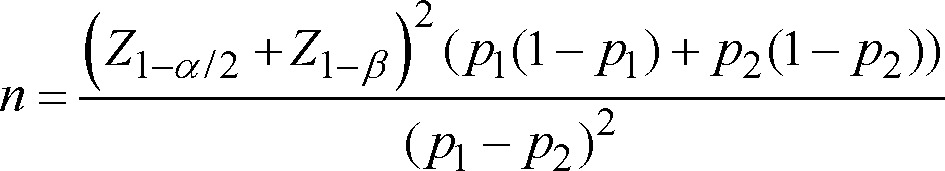


Because of our limitation in this study, we considered 24 patients in 1 group and in other group 19 patients entered in the study.

In this study, all patients with RRMS who were under treatment with low dose or high dose IFN and referred to the Neurology Clinics and/or were the member of the MS Society of Sanandaj in 2014 and met the inclusion criteria were enrolled in the study. Data were collected through questionnaires and interviews with patients and conducting MRI at the beginning and end of the project.

To conduct the study first, size and enhanced plaques on initial MRI were recorded. Then again MRI was done after a year, and the results were compared in terms of the number, size, and enhanced plaques. All MRI tests were performed in a specified imaging center. Drug side effects and relapse of disease were measured during the follow ups using questionnaires and interviews with patients and phone calls. 

The collected data were entered in STATA 11 (Stata Corporation, College Station, TX, USA) software. Chi-square and Fisher exact tests and logistic regression were used for analysis of data. The researchers in this study were committed to the principles of research ethics and observed research ethics issues for patients.

## Results

***Patients***

A total of 43 patients were enrolled in this study, and 24 patients (55.8%) were assigned to the group treated with low dose IFN and 19 patients (44.2%) were assigned to the group treated with high dose IFN. The mean age of patients treated with low dose IFN was 33.58 years, and the mean age of the patients treated with high dose IFN was 29.84; they had not a statistically significant difference (P = 0.073). Of patients treated with low dose IFN, 20 patients were female (46.51%) and 4 patients were male (9.30%). Of patients treated with high dose IFN, 15 patients were female (34.88%) and four patients were male (9.30%), and there was no statistically significant difference (P = 0.714). Table 1 shows demographic variables in 2 groups of study.

***MRI findings and relapse***

Based on the results of Fisher exact test, the P value obtained from this relationship (P = 0.039) was significant. In addition to the above test, we also used logistic regression. Based on the results of logistic regression analysis, compared with the low dose IFN therapy, treating patients with high dose IFN (with odds ratio of 5.19 and confidence interval of 2.1-32.4) had a better impact on the reduction of the number of plaques (Table 2). 

Table 3 compares the two groups in terms of the impact of drugs on the size of the plaque. During the course of treatment, six patients in each of the two groups suffered from relapse and the difference was not statistically significant (P = 0.633).

**Table 2:** Comparison of changes in plaque size based on the results of magnetic resonance imaging (MRI) in the two groups treated with low dose interferon (IFN) and high dose IFN

**Group**	**Reduction or stabilization in ** **the size of plaques [n (%)]**	**Increase in the size of ** **plaques [n (%)]**	**P**
The group treated with low dose IFN	17 (71)	7 (29)	0.059*
The group treated with high dose IFN	18 (95)	1 (5)	

* Fisher exact test. IFN: Interferon

**Table 3:** Comparison of side effects between two groups

**Side effect**	**Low dose IFN [n (%)]**	**High dose IFN [n (%)]**	**Ҳ** ^ 2^	**P**
Yes	21 (87.5)	18 (95.0)	0.658	0.417
No	3 (12.5)	1 (5.0)		

IFN: Interferon

***Side effects***

Of all, 21 patients in the low dose IFN group and 18 patients in the high dose IFN group had some degrees of side effects, and the difference was not statistically significant (P = 0.417).

Table 4 shows the relationship between the levels of reduction in the number of MRI plaques in the two groups. Based on the results of Fisher’s exact test, the P value obtained from this relationship (P = 0.048) was significant.

## Discussion

The aim of this study was to determine the effect of low dose IFN and high dose IFN and compare their effects on changes of demyelination plaques in brain MRI of patients with RRMS.

Based on the results of this study, compared with low dose IFN, high dose IFN was more efficient in stopping and healing patients in terms of the number of plaques. In addition, compared with low dose IFN, high dose IFN had better performance in stopping and curing patients in terms of the reduction in the size of plaques.

Our study has similarities with some other studies that have been conducted in this field. In Bastianello, et al.’s study,^14^ a total of 520 patients with RRMS were selected and were treated using IFN-β-1a with two different doses. The results showed that subcutaneous IFN-β-1a clearly played a role in reducing MRI plaques; in addition, using a higher dose was more effective in the treatment of patients and reduction of the plaques. This study is in line with our study as it showed that higher doses of the drug were more effective. Our study is also consistent with Mori, et al.’s study^15^ which showed that high dose IFN had a better impact on the improvement of some patients suffering from disorders caused by MS. The results of Lowery-Nordberg, et al.’s study^16^ also confirm our findings; they showed that high dose IFN had an impact on the levels of biological factors such as the PMP (CD31+) and PMP (CD54+) and it was also able to make changes in these markers.

The results of a study that was conducted by Hartung^17^ showed that treatment with high dose IFN is more effective for the prevention of relapse and it is the most important indicator. In a study by Schwid, et al.,^18^ which was conducted on the effects of IFN-β therapy in the management of relapsing MS, the results were consistent with the results of our study and showed that, compared with IM IFN-βa-1a 30 mcg QW, using SC IFN-β-1a 44 mcg TIW for the treatment of MS patients was associated with a significant reduction in clinical and imaging measures of disease activity over 1-2 years. In addition, the study also showed that patients who changed from low dose QW treatment to high dose TIW treatment experienced more benefits of treatment without a substantial increase in adverse events. The results of our study are different from Li, et al.’s study,^19^ as they reported that all types of IFN therapy can make changes in all parameters of the MRI. However, in our study, the difference in IFN dose was clear and that there were differences in the effects of high dose and low dose IFN.

In a systematic review study by Oliver, et al.,^20^ which investigated IFN-β treatments in adults with RRMS, the results indicate the high dose IFN therapy was more effective than lower doses in reducing relapse. This finding was not consistent with our results but it was in line with our study in terms of increased number of plaques and plaque stability.

**Table 4:** Comparison of reductions in the number of plaques based on the results of magnetic resonance imaging (MRI) in the two groups treated with low dose interferon (IFN) and high dose IFN

**Group**	**Reduction or stabilization in ** **the number of plaques [n (%)]**	**Increase in the number ** **of plaques [n (%)]**	**P**
The group treated with low dose IFN	14 (58)	10 (42)	0.048*
The group treated with high dose IFN	17 (89)	2 (11)	

* Fisher exact test. Based on the results of Fisher’s exact test, the P value obtained from this relationship (P = 0.048) was significant. IFN: Interferon

In a study by Prosperini, et al.,^21^ 121 patients with RRMS switched to high dose IFN-β and they were followed up for 2 years. The results of their study showed that switching from the low dose to the high dose IFN-β did not reduce the risk of further relapses or increased disability in the 2-year follow-up period. As a result, the findings of their study were different from ours. However, as in our study, they also recommended further studies to obtain more evidence.^19^ Unlike our results, in a study by Etemadifar, et al.^13^ no significant difference was observed between low dose IFN and high dose IFN in terms of the reduction in disease relapse.

As one of the limitations of this study, although clinical trial with randomization is the best way to test the hypothesis of this study, due to budgetary limitations and the long duration of the project, it became difficult for the researchers to use this method. A lack of implementation of clinical trials for this study may result in estimation errors. It is recommended to conduct clinical trial studies to investigate the effect of different pharmaceutical brands.

## Conclusions

This study has three key messages: At first, the two drugs were similar in terms of reducing disease relapse and complications (including flu-like symptoms, injection site reaction, injection site redness, and slight increase in liver enzymes). In addition, both drugs were effective in controlling active and demyelinating plaques and preventing the activation of plaques. However, high dose IFN-β-1a was more effective in reducing the number and size of MRI plaques in patients with RRMS. Second, it is recommended to conduct more properly designed clinical trials. To better assess the effects of low dose IFN drug especially for Iranian-manufactured IFNs, it is recommended to carry out similar studies with more patients and with a longer time periods to assess the reduction of disability, relapse, and complications and to evaluate the improvements in the results of brain and spinal cord MRI; such studies can also assess the effect of the time of initiating treatment process. Third, as a practical suggestion, it is recommended to use high dose IFN-β-1a for RRMS patients as high dose IFN-β-1a drug is more effective in reducing the number and size of MRI plaques.

## Notes:

**How to cite this article:** Khomand P, Moradi G, Ahsan B, Abtahi S. Comparison of the effects of low dose interferon and high dose interferon on reduction of the number and size of plaques in patients with Multiple Sclerosis: A historical cohort. Iran J Neurol 2017; 16(1): 1-6.
